# Using social media research in health technology assessment: stakeholder perspectives and scoping review

**DOI:** 10.1017/S0266462323002593

**Published:** 2023-09-21

**Authors:** Anke-Peggy Holtorf, Andriy Danyliv, Li-Ying Huang, Yvette Venable, Alissa Hanna, Annekatrin Krause, Miranda Pierre, Donna Walsh, Aline Silveira Silva, Sou-Hyun Lee, T. Joseph Mattingly

**Affiliations:** 1 PCIG at HTAi, Project Coordinator, Basel, Switzerland; 2 College of Pharmacy, University of Utah, Salt Lake City, USA; 3 Patient Engagement, Novartis Pharma AG, Switzerland; 4 Division of Health Technology Assessment, Center for Drug Evaluation, Taiwan; 5 Patient Engagement, ICER, USA; 6 Patient Engagement, Edwards Lifesciences, USA; 7 Scottish Medicines Consortium, Healthcare Improvement Scotland, Glasgow, Scotland; 8 European Federation of Neurological Associations, Ireland; 9 Patient Voices Network, Brazil/Canada; 10 University of British Columbia, Vancouver, Canada; 11 NECA, South Korea

**Keywords:** social media research, social media listening, health technology assessment, patient-based evidence, stakeholder participation

## Abstract

**Objectives:**

The aim of this initiative was to examine collaboratively, in a multi-stakeholder team (health technology assessment (HTA) practitioners with patient involvement expertise, health technology industry, patient advocates, health policy experts, patient engagement experts), whether evidence generated through social media research (SMR) fills current information gaps relating to insights on specific aspects of patient experiences, preferences, or patient needs and delivers additional value to HTA.

**Methods:**

The framing of the project was done in a co-creative, deliberative multi-stakeholder process. Challenge and refinement happened through discussions with 25 independent stakeholders from HTA bodies, industry, academia, and patient advocacy. For critical themes identified during the framing phase, scoping literature reviews were performed including the state of methods and examples for the use of SMR in HTA.

**Results:**

The framing and stakeholder discussions specified a set of expectations and requirements, and the scoping reviews revealed the current state of methods and usage of SMR in health-policy decision making.

**Conclusions:**

The project concluded that SMR can contribute new, relevant evidence to HTA. It is however recommended to evolve the science through defining best practices when planning, conducting, and using SMR and to conduct multi-stakeholder pilot SMR projects to address questions relevant to current HTAs and to validate and improve the proposed practices.

## Introduction/Background

In health technology assessment (HTA), the determination of value is dependent on the dimensions and the perspectives considered (1). The patient perspective is one important aspect of HTA and can be represented through the direct involvement of patient representatives or through review of patient-based evidence (2). It is recommended to perform robust research into patients’ needs, preferences, experiences, and patient participation for including the patient perspective in value determination. However, it is not yet well defined how such research can be integrated into the specific national or regional HTAs to fulfill the high evidence expectations. Some HTA bodies rely on participatory processes through surveys, templates, hearings, or representation by patient organizations in HTA committees to account for the patient perspective (3–5). In contrast to evidence that can be critically assessed, patient input arising from participation tends to be more topical, able to provide additional insights during any deliberation throughout the HTA, and is generally strong in reflecting the local context such as service variation or special access conditions.

While participatory processes for involving patients in HTA are evolving and are applied in various forms by HTA bodies (3; 4), limitations of the current involvement procedures have also been noted (5–8). The complexity and individualization of involving patients puts a high burden on the resources of HTA bodies as well as of patient organizations or patient advocates. Dedicated, well-trained personnel as well as the capacity for timely delivery of the information requested by the HTA body are needed. In current frameworks, patients may be underrepresented due to location, symptoms or severity of illness, language, cognitive function, length or progress of illness, rarity of disease, or lack of recognition particularly if they are not represented by a patient organization. Finally, the uncertainty of the representativeness of individual qualitative input somewhat devalues it in comparison to the quantitative data-based evidence.

Several approaches are currently used in HTA to integrate the patient perspective including studies of Patient Reported Outcomes in the context of clinical studies, Patient Reported Experience studies, or surveys collecting information on real-life experiences relating to the disease or the delivery of care (3). It has been suggested that an additional solution to improve the representativeness and comprehensiveness may be non-interventional, observational analysis of ‘natural’ communication between patients or caregivers in social media (9). As patient and caregiver communities are open to broad participation from all levels of society, and are often used to discuss own experiences, seek help from people in a similar situation, and get advice for the daily living or coping with one’s own health or disease, the communication in social media may be a wider source for understanding patient experiences in a more representative, qualitative or quantitative manner than through direct active input from individuals or selected populations through testimonies, interviews or surveys. Research on social media, henceforth referred to as Social Media Research, such as social media listening or sentiment analysis, may yield insights into patients’ needs, expectations, and experiences from a wider group of patients in a more standardized or objective fashion being less dependent on selective individual experiences (10–14). On the other hand, Social Media Research may introduce new biases such as relying on input from digitally active patients who may predominantly discuss negative experiences or from sources aiming to manipulate the discussion among users (13; 15).

While some attention has been given to the detection of infective diseases or adverse events through Social Media Research, little work has been done yet on its use for HTA purposes. Street and Farrell suggested that analysis of patient communication in social media may help to retrieve information on patient experiences in “hard-to-reach” patient types and that social media may be a more rapidly accessible source of information on patient experiences (9). However, they also caution that important ethical and methodological challenges with collecting patient views and experience via social media need to be addressed.

To fill in the gap in understanding the conduct of Social Media Research and its use in HTA, Patient and Citizen Involvement Group of Health Technology Assessment international (HTAi; www.htai.org/pcig) initiated a multi-stakeholder project to explore whether Social Media Research can generate evidence which fills current information gaps relating to patient experiences, patient needs, or patient values and can deliver additional value to HTA.

This initiative driven by a team of diverse stakeholders with patient involvement expertise (HTA practitioners, industry, patient advocates, health policy experts) aimed to collaboratively examine and describe the current environment related to the use of *Social Media Research* in HTA.

## Methods

### Definitions

***Social media*** are mobile and web-based highly interactive platforms via which individuals and communities share, cocreate, discuss, and modify user-generated content (16).

For the purposes of this report, ***Social Media Research*** is defined as research with data originating from any social media platform including, but not limited to: Twitter, Facebook, YouTube, Instagram, Reddit, blogs, and chatrooms or forums (closed, password-protected, or open, non-password-protected). The research may refer to large quantitative data mining/modeling methods through to more qualitative in-depth analyses. *Social Media Research* generally aims to reveal new insights into information sharing or policy discussions, to understand personal experiences and opinions or sentiments from the individual or patient community perspective, to conduct epidemiological studies, to identify and detect adverse events or other clinically relevant reports, or to observe online behavior.

### Project process

The PCIG subcommittee (core project team) consisted of patient advocates and patient engagement experts from HTA agencies (4), patient organizations (3), academia (3), and industry (2 pharmaceutical, 1 medical devices).

#### Step 1: Framing the issue

The project started with a framing workshop. Informed by the respective expertise and experience of each of the participants, the PCIG project team defined in collaborative work sessions using an Online Collaboration Whiteboard the expected deliverables, properties, and requirements for the use of evidence generated through *Social Media Research* in HTA from five perspectives: Patients, HTA agencies, Research, Industry, and the Public. The resulting whiteboard contributions were clustered by themes (grounded theory approach), discussed, and then revised based on the consensus in the discussion.

The different perspectives were challenged and complemented through individual discussions with independent stakeholders including experts from research (1), HTA agencies (5), industry (5), or patient organizations (14) during the time from March to June 2021 following a discussion guide (Supplementary File 1). All patients were members of patient organizations and had various levels of experience with social media as moderators or users. The independent stakeholders were selected from a long list of contacts that were proposed by the team members after prioritization to achieve a broad coverage of stakeholder types, healthcare systems, disease areas, and age groups for the patients. The final selection was determined by the willingness to participate in these discussions.

The inputs relating to the acceptability, the perceived risks, expected governance, and feasibility of using *Social Media Research* for informing HTAs were recorded after each discussion in an Excel spreadsheet and summarized for each stakeholder group by one reviewer.

#### Step 2: Literature reviews

Three subjects emerged from the stakeholder input in the framing phase that required a more in-depth review of the current state of the art, and which were therefore explored through scoping literature reviews:

- Robustness of *Social Media Research* Methods
- Case examples for *Social Media Research* in healthcare decision making
- Privacy / Legal / Ethical Considerations (reported in a separate publication (17))

For each of the scoping reviews, a list of specific keywords was defined by the three members of each review team and then applied to a search in CINAHL, PubMed, EMBASE, and/or Google scholar. The retrieved long list of articles was screened by title for inclusion or exclusion by the review team members. This resulted in the inclusion of 41 (Privacy / Legal / Ethical Considerations), 75 (Social Media Research Methods), and 23 (Use of Social Media Research in Decision Making) papers, respectively. Each team member extracted relevant information according to agreed-on criteria. The overall scoping summary was consolidated through a consensus of the respective three team members.

The entire process and findings were summarized and discussed in a combined final report, which was circulated for consultation in the PCIG community and to selected stakeholders who had not been involved in the project before. The report, revised after the consultation, serves as a foundation for this manuscript.

## Results

### Expectations for social media research for HTA purposes

The co-creatively defined stakeholder expectations and requirements for Social Media Research guided the subsequent project flow including their validation through discussions with independent stakeholders and the three literature reviews. The stakeholder perspectives and expectations are summarized in Table 1.

**Table 1.** Stakeholder perspectives and expectations for social media research or a social media research platform as co-created in two team workshops and refined through stakeholder discussions

Broad access to patient experience/one recognized source for all	Patient organizations/patient advocates Accessible for research by patient organizations Health technology assessment bodies The system can systematically reveal patient-relevant outcomes, expectations, preferences, experiences, unmet needs, and impact of disease or treatment on their life The system allows to reveal subgroup divergence The system allows for cross-country comparison The research follows a consistent definition of patient experience and preference data, which is transparent across HTA’s and based on current best practice Ability to retrieve hard to find patients (or communications from these patients) Industry Evidence-based approach for research on patient needs which is aligned across stakeholders Operational model of social media research for generating patient insights, which is accepted by patients and HTA bodies Credible base for discussing and defining HTA expectations for COA and PROMs Allows for access to patient insights earlier in R&D decision making to prioritize R&D along patient needs Credible source for identifying the unmet needs of the patient in alignment with HTA process Research/academics Allows for methodological research such as linking social media research to PROs The research approaches help to overcome the limitations of current methods, e.g., individual patient input, patient testimonies Access to rich but unstructured data and development of suitable research methods Public/citizens A framework & principles created by multi-stakeholders that can be accessed, but not manipulated, by the public
Stakeholder interaction	Patient organizations/patient advocates It is clearly defined how patient organizations are involved (engaged) in the project, research, and data collection throughout the project Health technology assessment bodies The system will be an additional source of information and no replacement for engaging with patients
Language/terminology	Patient organizations/patient advocates Handles data in lay language (colloquial evidence) Retrieving and analyzing unsolicited, natural conversation about how patients are experiencing their disease or health state Recognizes and reports patient terminology Linking to professional terminology Public/citizens Supporting the formation of a linguistic interface between patients, science, medicine, and public
Protection of users & data privacy	Patient organizations/patient advocates Data privacy is fully respected by only using content, which is publicly accessible or volunteered dedicated access for the research Public/citizens Ethically acceptable Non-hazardous
Retrieve information representatively	Patient organizations/patient advocates Finds the patient conversations (instead patients having to find the platform) No additional work for patients; transparent and reflective of real patient needs Research/academics Clarity around representativeness of data (patient type inclusion etc.)
Trust, validation, credibility	Patient organizations/patient advocates The purpose, benefits, and limitations of insights derived from social media research are clearly defined The insights derived from social media research reflect real-life and current patient needs & challenges Health technology assessment bodies Transparent definition of agreed-on process Ensures credibility of input/output data Face validity to be confirmed Public/citizens Reinforces assurance that resource allocation is happening with patients’ needs in mind Legitimacy of HTA recommendations and HC decisions
Transparency/feedback	Patient organizations/patient advocates The results are reported openly and accessible through the website or an affiliated website The system is transparent and there is a feedback service or mechanism Research/academics Transparency (and mitigation) of limitations, e.g., missing all patients who are not using social media Public/citizens The process, methods, results, and use of results are transparent
Reference to healthcare reality	Health technology assessment bodies Enable/facilitate definition of relevant outcomes/study endpoints Can reveal patient perspective on the impact of a new technology on their life Identifying trends in specific disease areas Continuous observation (reference) of changes in disease area as communicated by patients/caregivers Serves as colloquial evidence database, patient information source for HTA Industry Source for identifying new opportunities of unmet patient needs helping to develop new/improved solutions to address those needs
Relevance	Health technology assessment bodies The results of social media research must be relevant and an improvement to HTA criteria & questions
Methods, robustness	Health technology assessment bodies Systematic information Research validation & result validation Addresses uncertainty Industry Validated research approaches Clear guidance/best practice for generating patient insight evidence Research/academics Guidance on sources of bias with social media research and how to mitigate the bias Clear structures and governance
Scientific recognition	Industry Opportunity to publish and enhance research with a focus on patient needs, experiences, preferences Research/academics Allows for research with a broad range of data on patients’ needs, experiences, and priorities Allows for publishing primary (observational) research on patient experience and preferences in uncensored colloquial evidence database (social media)

Abbreviations: HTA, health technology assessment; R&D, research and development.

The main expectations formulated by the stakeholders were that *Social Media Research* can systematically reveal patient-relevant outcomes, expectations, preferences, experiences, unmet needs, and impact of disease or treatment on patients’ life without putting additional burden on patient communities. Thereby, it is expected to form a source for identifying the unmet needs of the patient in alignment with HTA process, which can inform HTA researchers on a variety of aspects including the suitability of the COAs or PROs (to be) used in clinical trials, subgroup or regional divergence, changes of patient experiences over time, or matching of terminology used by patients and professionals (linguistic interface). The advantages expected from *Social Media Research* include that the research finds the patient conversations in their own language (instead of patients having to find the platform or survey or other means of contributing to the HTA process and learning the vocabulary applied there), that it does not create additional work for patients, and that it allows for continuous observation of changes in disease area as communicated by patients or caregivers.

On the other hand, the strongest concerns emerging from the stakeholder perspectives mainly related to two topics: (i) Legal and ethics requirements including privacy protection, and (ii) anticipated risks related to the data and research robustness.

Within the Legal and Ethical Requirements, the consideration of existing ethical frameworks and legislation for *Social Media Research*, the handling of data from different types of sources, and security issues seemed particularly important.

Anticipated research and data-related challenges included data biases or robustness of methods, potential abuse of system or data, manipulated or fraud data, validity of the evidence, and resource- and scope-related challenges such as lack of time and expertise in HTA agencies.

### Stakeholder discussions

Individual discussions were held with 25 stakeholders representing different roles or perspectives in the HTA process (5 from HTA agencies, 14 patient advocates, 1 academic, 5 from industry). The summary of the results of all discussions is included in Supplementary File 2.

The stakeholders generally confirmed the expectations outlined originally by the project team. Only few additional expectations or concerns were raised. Specifically, both patient and industry advocates emphasized that any guidance, data, and analytics should be accessible to all stakeholders including patient organizations, academic research, industry, and HTA researchers.

Likewise, patients and industry postulated that with acceptance of best practices for *Social Media Research*, HTA agencies should also formally accept this evidence as admissible for HTA or in specific HTA process steps (e.g., early scientific advice, discussion of relevant clinical trial endpoints, lifecycle management).

Across the board, the stakeholders advocated that evidence generated by *Social Media Research* is only seen as additional complementary information and should not replace any of the current forms of evidence or input. The findings should always be validated through other means.

On the other side, all stakeholders were concerned, that currently there is only inadequate and unsatisfactory protection in *Social Media Research* against manipulation or abuse of the system (e.g. snooping, astroturfing, misrepresentation of certain populations, false sites, disinformation, content or research generated through artificial intelligence, etc.).

Patient advocates were unsettled about using closed-groups communication without the consent of the participants. They warned that even if intimate information is openly shared among patients, some patients may not want to share personal information for research purposes.

HTA practitioners cautioned that HTA agencies typically neither initiate nor conduct new research but mostly review and appraise the evidence published or submitted in dossiers by the innovator or by patient organizations.

#### Credibility of social media research for HTA

All stakeholders saw potential value in *Social Media Research.* Patient advocates identified validation by other types of evidence (e.g., testimonials, surveys) as an important complement to strengthen the credibility of *Social Media Research.* The HTA stakeholders were more hesitant and emphasized that *Social Media Research* would always be assessed in specific contexts for its potential value. Such research could be used to identify themes and inspire new ideas, with the intention of follow-up with more robust methods to further explore those themes. For example, results from *Social Media Research* could inform the early scientific advice for certain health technologies or the initial phases of an HTA (scoping).

There was a high awareness that *Social Media Research* is already being performed in academic settings as well as driven by industry, platform owners, or patient organizations. Current use of *Social Media Research* as recalled by the stakeholders included info-demiology (‘internet-epidemiology’, the science of distribution and determinants of information in an electronic medium, specifically the Internet, or in a population, with the ultimate aim to inform public health and public policy (18; 19)), info-veillance (public health surveillance through internet-based data sources containing unstructured information from multiple origins (18; 20)), pharmacovigilance, applications in market research, or sentiment research.

#### Opportunities and risks of social media research for HTA

Possible advantages or opportunities of *Social Media Research* were identified by all stakeholders and are summarized in Table 2. On the other hand, several risks or concerns related to *Social Media Research* are reported in Table 3.

**Table 2.** Perceived advantages of social media research identified by stakeholders in the framing phase

Identify unmet needs	Help exploring unmet needs in an unsolicited way Early information for HTA practitioners on patient perspective
Find hard-to-reach patients	Patients with rare diseases or their caregivers Diseases without any formal or active patient representation
Guide understanding of how patients experience a disease or a therapy	Gathering directly patient and caregiver level of information Identify context of disease and therapy experience *(Particularly at onset of HTA or for Early Scientific Advice to inform PICO)*
Unsolicited & bottom-up research	Capturing perspectives not always captured by patient organizations Increase diversity of patient perspectives Mapping the disease Journey
Efficiency	Faster than prospective, interactive research (e.g., surveys, interviews) or clinical studies Less resource intensive as other types of research Wider reach with reduced burden to patient organizations
Confirmatory	Compliments other data, observations, or reports Increased evidence for business case for a product
New healthcare opportunities	Opportunities for preventive care Observation of impact of certain policy interventions and perceptions in the public or patient community Sentiment analysis to address fears, resistance, community driven behaviors or schemes
Crowd learning for patients (limited to interactive Social Media Research)	Increased sharing of knowledge from patient to patient Increased social support and build relationships

Abbreviations: HTA, health technology assessment; PICO, patient–indication–comparator–outcomes.

**Table 3.** Perceived risks of social media research identified by stakeholders in team and stakeholder discussions

False input data	Fake posts (e.g., astroturfing), manipulation Input driven by discussion impetus or misinformation rather than by patient experience Impact of harassment / bullying
Lack of consent	Possible issues of consent (e.g., patient would not have agreed if asked even though the discussion was public) Inappropriate use of data “Snooping” & intruding privacy
Bias	Some patients may be under/overrepresented Risk of manipulation Risk of selective reporting
Methods	Complexity of research Tracking the wrong thing Lack of data and result consistency Separating noise from signal
Ownership of data	Public versus private communication Social media platform provider versus individual contributor vs researcher
Representativeness	Lack of context Not representative
Equity/access	Overweighs the perspective of those with access and high level of engagement in social media Limited applicability for some diseases
Continuity/follow-up	Selective engagement, e.g., reduced engagement after patient is cured
Justifiability	Balancing information need (benefits) versus intrusiveness
Governance	Interests of the research sponsor / platform owner Selective reporting determined by stakeholder interests

### Scoping reviews

#### Social media research methods

Of 135 references of potential interest (published between 2016 and April 2021), 75 were included in the analysis. The key findings from the analysis are summarized in Table 4 and, more detailed, in the online Supplementary File 3.

**Table 4.** Summary of findings of the literature search on methods for social media research (N = 75)

Data sources	Mainstream social media sites: e.g., Facebook, Twitter, Instagram, or YouTube Data collected in proprietary sites (e.g. PatientsLikeMe) Data from subject related blogs Types of communication can include written text, spoken text, or videos.
Study types	Commentaries 16% (12/75) Cross-sectional analysis 14.7% (11/75) Reviews 13.3% (10/75) Most of the research was observational and a few studies included surveys or other forms of interaction. Surveys (as often used by patient organizations) were not part of this project but only observational studies of the self-initiated communication without interfering or guiding the direction of the communication.
Methods	Sentiment Analysis using Natural Language Processing [NLP], computational linguistics, information retrieval, and data mining techniques, Artificial Intelligence (AI), Machine Learning (ML) or deep-learning techniques or lexicon-based manual browser analysis to classify the emotional connotation or positive / negative opinions in unstructured free text. Many standardized analytic tools are proprietary and exist at cost; free NLP frameworks have also been developed and are openly available. Supervised and unsupervised approaches depending on the data source.
Applications	***“Info-veillance”***: Public health surveillance based on the continuous and systematic collection, analysis, and interpretation of social media data serving as early warning systems to monitor epidemics or to document the impact of interventions. ***“Info-demiology”***: Detection of emerging disease epidemics, seasonal changes in demographics, comparison of disease / Treatment searches across countries or regions, detection of flaring infections, exploring changes and trends over time ***Pharmacovigilance***: identify serious, unknown, or unexpected adverse drug events ***Public perceptions***: Sentiment analysis to better understand public perceptions and opinions on specific healthcare policies or interventions ***Patient need identification*:** To gain better understanding of patient needs and to inform the choice of study endpoints in clinical trials or the product features and development
Limitations	**Manual, lexicon-based techniques**: clean classification may be challenging and depending very much on the individuals doing the analysis; very time consuming and error prone **Country- or context-specific analyses** is often restrained by privacy settings that limit geo-localization of data Without **user identification**, separate posts coming from the same individual cannot be identified as duplications and may skew the results. **Limited comparability** of results generated with **different methods.**
Ethical review	In only 9.3% (7/75) of the studies, ethical review was reported.

Some potential use cases for *Social Media Research* with insights useful for HTA were reported such as info-veillance, info-demiology, pharmacovigilance, public-perception research, and patient need identification. Most of the current *Social Media Research* is observational in nature and is assisted by some form of advanced analytic techniques to avoid resource-intense manual browsing and identification. Some challenges related to the accuracy of automated procedures for *Social Media Research*, data pattern identification, as well as data sourcing remain to be addressed. Due to the seemingly public nature of the data as well as novel research pathways, ethical review is rarely done – an area that warrants further guidance.

In addition, such research is done by a range of researchers with diverse professional backgrounds and value frameworks such as IT-related sciences, clinical research, or behavioral/social sciences.

Our findings are consistent with the recommendations from other recent reviews (21; 22), which concluded that “the outcomes of these applications should be considered exploratory” (in the context of sentiment analysis) (22) but also suggested that the current body of research should be used to inform further development of more structured and consistent methods as well as guidance (21). Therefore, a second, separate output from this project is an initial guidance or framework to support future producers and users of *Social Media Research* in quality assurance published in this same journal (17).

However, constant updating will be needed to cater for new developments. For example, some of the limitations listed in Table 4 are already being addressed through newer approaches such as improved recognition of fake reports (23) or using pretrained models. Nevertheless, despite advanced methods, diligence in external validation and model re-calibration before implementation are still recommended for ensuring clinical meaningfulness (24).

#### Use of social media (research) in HTA

Of 580 potentially relevant references from the years 2016–2022, 23 were retained for inclusion after screening of title, abstract, and full text. Key findings of the analysis are summarized in Table 5. A more in-depth report is available in the Supplementary File 4.

**Table 5.** Results of the literature review on case examples of the use of social media research for HTA or healthcare decision making (n = 23)

Countries/regions	Sixteen studies included data from the USA (16; 70%) or addressed US-specific questions and five studies had a global scope (5; 22%), the UK was included in three studies (3; 13%), France in one (1), and several European countries in one (1)
Policy level/organization	The studies were initiated by regulatory authorities (13; 56%), by industry (4; 17.4%), public health institutions (3; 13%), academic institutions (13%), or the platform owners (2; 8.7%) with the purpose of informing health policies, clinical endpoints choices, methodological questions, or with promotional intentions or demonstration of feasibility.
Intentions	Inform the clinical trial design and endpoints, Understand health impact perceptions, Testing the suitability of methods for informing healthcare policies, Increasing the awareness of the platform opportunities for this type of research.
Healthcare area	Adverse event detection or pharmacovigilance (8) Sentiments / behaviors relating to Tobacco or Marihuana consumption (3) Disease or healthcare focus: COPD (1), Dry Eye Disease (2 by the same researchers), Cardiac safety (1), Ritalin (methylphenidate) (1), Diabetes (2), Acute Myeloid Leukemia or Myelodysplastic Syndrome (1), Statins (2), Opioid Use Disorder (OUD) (1), Pulmonary Arterial Hypertension (PAH) identification (1), Surgical hernia mesh public perceptions (1), HPV vaccination risk perception (1), Off-label drug use (1), Perceptions of CDC in the public COVID-19 related discussions (1) Food safety–related incidents and recall related public perceptions (1)
Social media type/data sources	Most frequent: Twitter (11), Facebook (7), Patient / Health forums (6), general unspecified data (5), and Inspire (4) Less frequent: Instagram (2), blogs (2), Newswires (2), Reddit (2), unstructured FDA data (2), and public dockets (1); French open discussion forums (1): www.atoute.org, www.doctissimo.fr, www.e-sante.fr, www.onmeda.fr (previously www.aufeminin.com), sante-medecine.journaldesfemmes.com.

Abbreviations: COPD, chronic obstructive pulmonary disease; CDC, center for disease control; FDA, food and drug agency.

No specific case was identified within the time frame of the search where *Social Media Research* has directly impacted HTA.

However, social media discussions have impacted healthcare-related decisions in not-evidence-based ways: Fadaee et al. warned that interest-driven negative anti-hernia mesh communication in social media -- largely driven by lawyers in the USA (three of the five top tweeters were linked to law firms involved in mesh-based lawsuits) -- might provoke a negative FDA decision, as it has happened before with pelvic/vaginal mesh (15).

The FDA, academic researchers, and the European WEB-RADR consortium have started to use *Social Media Research* for adverse event detection. WEB-RADR concluded that methods and data are not yet robust enough for standard use in pharmaco-vigilance (25) and strongly recommended to always corroborate findings from *Social Media Research* through other means.

In other examples, sentiment analysis revealed information on people’s attitudes and motivations with the purpose of informing public health policies or communication. Pharmaceutical industry has used *Social Media Research* to better understand patient experiences and needs and to inform the early dialog processes with HTA agencies for the choice of patient-relevant endpoints (13). Although the data may have been included in the submission dossiers for reimbursement, how these data are in fact used by different HTA agencies, is unclear.

Many potential applications of *Social Media Research* in healthcare policy-making, −implementation or -communication are emerging, but not much of this emerging evidence is currently routinely used in HTA and no example has been identified, where HTA agencies have commissioned *Social Media Research.*

## Discussion

For the question asked at the outset of this work, there seems to be a broad consensus that *Social Media Research* has the promise and potential of revealing new and complementary information about patient experiences, needs, and behaviors. This evidence may help to test the potential benefit of new health technologies to the patients, which may in turn help to make more patient-relevant decisions about health technologies (26).

However, *Social Media Research* typically depends on secondary use of private communication data. Due to the rapid developments in the pathways of communication in social media and likewise, the means and methods for research using these data, not all risks to the data producers or the people they are communicating about are known. Special care should hence be taken for their protection throughout the lifecycle of *Social Media Research.* More detailed guidance emerged from the literature review of the ethical and legal aspects of *Social Media Research* and is published separately (17).

Since there are high expectations for the value and use of *Social Media Research*, it appears appropriate for the HTA community to proactively engage in the creation of a framework in which *Social Media Research* can be considered and used within HTA.

However, due to evolving methods for retrieval and analysis and the changing properties of the data sources no best practice has been defined yet. Examples of the use in policy and decision making are rare and currently, HTA agencies seem to be curious but careful if not hesitant in utilizing such evidence when evaluating new technologies. Reasons for such hesitancy are manyfold (e.g., lack of competence for qualitative research in the assessment team, current local HTA regulation driven by almost exclusive consideration of RCT-based evidence in some countries, distrust in robustness of methods, distrust relating to lack of justice and representativeness, distrust in quality of data, etc.). Reliable means are needed to determine the robustness of methods and meaningfulness of the results and thereby, facilitate using the evidence from *Social Media Research* for HTA. These should be addressed in a future guidance for researchers and HTA practitioners on *Social Media Research* to gain more experience with this type of evidence and address the potential resistance. An attempt to initiate such guidance is published separately in the same issue of this journal (17).

At this stage, however, considering that the methods, means, and data for such research are constantly refined and advanced, with continuing uncertainty around their robustness, it seems premature to develop a standard approach and hence, unlikely to happen soon. In contrast, we advise an individual ethical review of each research concept and plan to judge whether the expected benefits of the research will outweigh the potential risks or harms to those who have communicated on the social media (17).

Despite the increasing automatization of methods, most of the examples reviewed for this report still involve large amounts of ‘manual’ work which requires considerable expertise and resources. While HTA researchers are intensively trained for assessing and appraising clinical trial-based data and studies, they may be less experienced with qualitative data analysis and evidence (6; 8).

Consequently, future work should focus on guiding (i) *Social Media Research*, (ii) the use of the evidence originating from *Social Media Research* in HTA, and (iii) on learning from the experiences with the evidence generated through *Social Media Research.*

## Limitations

Only limited resources were available for this initiative and the report can only be seen as an approximation to the subject rather than providing a final assessment. With increasing experience in *Social Media Research* and its use for guiding policy making, new opportunities might be recognized, which were not included in this work.

The scoping reviews are pragmatic and not exhaustive. However, sufficient saturation was reached in all three themes of the scoping reviews to inform and guide the recommendations provided by this report.

There was no expert on ethics or legal aspects among the team members. Due to the importance of these issues, it is recommended to include such experts in any future work. In addition, although being a multi-stakeholder team, the representation of some stakeholders was limited due to the small size of the team. For example, there was only one full-time academic researcher on the team. However, we hope that this paper is a starting point to bring more researchers (in all their diversity) in and start working intensively on this urgent subject, together with other stakeholders.

The work in this report mostly focused on the use of *Social Media Research* for assessing new technologies and less on the use of *Social Media Research* to inform the priorities or communication strategies of HTA agencies that aim at improving public and patient acceptance and perceptions of the value of HTA.

## Recommendations

Best practices and a framework should be defined to help researchers, patient communities, and HTA practitioners or committees to ensure and determine the credibility of the results of *Social Media Research* in the context of a specific HTA.

To initiate this, a separate report proposes a set of principles for planning, conducting, and reporting the results of *Social Media Research* aiming to maximize the relevance and applicability and, in addition, to allow for assessing the legal and ethical integrity and protecting the individuals who communicate about themselves or are affected by the communication of others (17).

In addition, pilot programs including the planning, conduct and reporting of a *Social Media Research* study addressing a subject of interest to one or several HTA agencies should be organized in a multi-stakeholder consortium to test and improve the guidance initiated with this project. Stakeholders who should be part of such a consortium include HTA practitioners, case-relevant patient organization representatives, consumer representatives, academic researchers in outcomes research and health economics, methodological-analytical experts, and experts in patient-based evidence as well as ethicists.

## Supporting information

Holtorf et al. supplementary material 1 Holtorf et al. supplementary material

Holtorf et al. supplementary material 2 Holtorf et al. supplementary material

Holtorf et al. supplementary material 3 Holtorf et al. supplementary material

Holtorf et al. supplementary material 4 Holtorf et al. supplementary material

## Supplementary material

The supplementary material for this article can be found at http://doi.org/10.1017/S0266462323002593.
