# Supplementary material for: Using social media research in health technology assessment: stakeholder perspectives and scoping review
Source: Int J Technol Assess Health Care. 2023 Sep 21;39(1):e63. doi: 10.1017/S0266462323002593 (PMC11570010; doi:10.1017/S0266462323002593)
Supplement: Holtorf et al. supplementary material 1 — Holtorf et al. supplementary material [file S0266462323002593sup001.docx]

Perceptions of Patient Insight Research Platform (PIRP)

Discussion Guide

| Name: |  |
| --- | --- |
| Organisation: |  |
| Function: |  |

| **Stakeholder Perspective:**  Patient Organization  HTA Expert  Pharmacist / Medical Doctor  Public  Sciences / Research  Industry: _____________  Other: _____________ | **Country / Region**  _________  _____________ |
| --- | --- |

## Flow of the discussions

1. Short summary presentation of what the PIRP shall be and what the purpose of our project is (the Description of the PIRP requirements and outline of a pilot)
2. Consent
3. Structured discussion using a uniform interview questionnaire

## Consent

The purpose of the following discussion is to consult on our current outline of the PIRP project and whether it reflects your perspective on the creation of a Patient Insight Research Platform (PIRP) sufficiently. Your responses may be used to strengthen the stakeholder viewpoints and to refine the concept, which we plan to publish as a whitepaper either to the HTAi community and to an audience beyond HTAi ([www.htai.org](http://www.htai.org)).

The results will only be used for this purpose and will not be made available to a broader audience.

Do you agree to participate in these interviews and that your responses are used anonymously as described above?

Yes / No Date :

# SECTION 1

1. Do you agree with the expectations for PIRP from your perspective?
   *(Looking at the bullet points from the respective perspective)*
2. Are there additional expectations that should or could be met?
3. Which additional stakeholders and should be considered / involved?
4. What kind of governance would you expect for a PIRP (ownership, hosting, oversight, etc.)?
5. Who, in your opinion, would have an interest in manipulating the PIRP (e.g. hacking the data or algorithms) and from your perspective, what is the level of risk?
6. Which level of transparency do you expect from your perspective?
   *(e.g. data only accessible for HTA bodies vs scientific publications vs access to patient community / public vs report all results on dedicated website)*
7. In your opinion, do you feel a PIRP could be a credible source for identifying unmet patient needs?
8. Are you aware of any practical example in which social media data was used to generate patient-based evidence?

# Section 2 (specific for each stakeholder type)

## Patient community

*(Consider from both, the perspective of the individual patient or the patient advocates)*

1. What is the current understanding / knowledge level within the patient community related to social media research approaches?
2. How do you feel about using data from social media for research and evidence generation on patient needs and experiences?
3. Where do you see the advantages / opportunities of using such an approach?
4. Do you see any potential downsides or risks to patients or the patient community?
5. Does this approach have different implications for individual patients vs the patient advocacy community?

## HTA Agencies / Experts

1. Are you aware of any regulations on the use of social media from your agency (from the legal ethical perspective)? (References?)
2. Where do you see the advantages / opportunities of using such a platform?
3. Which risk or downside in using such a platform do you see?
4. What would help to overcome some of the challenges (probe for specific challenges)?

| Data privacy |  |
| --- | --- |
| Methodology |  |
| Validation of results |  |
| Data bias* |  |
|  |  |
|  |  |

**(e.g. not reflecting patients who are not communicating social media about their health-related experiences)*

1. In your opinion, what do you think is the added value of such a platform? *(e.g., how could a PIRP fit within the existing HTA agency process and strengthen it?)*
2. How would you determine if data generated from social medial research is credible?
3. Where should the platform be hosted & managed to be useful, trusted, and used by your agency?
4. How could the maintenance of such a platform be financed and resourced?
5. Any other prerequisites which we need to consider?

## Research / Academics

1. Where do you see the advantages / opportunities of using such a platform?
2. Which risk or downside in using such a platform do you see?
3. How do you assess the feasibility of creating a fully functional PIRP?
4. Are you aware of any guidance on the appropriate use of social media for research (from the legal ethical perspective)? (References?)
5. Is there methodological guidance / best practice for SMR? (References?)
   *(including methods how to handle uncertainty / bias)*
6. What needs to be done for validation of the methods and the results?
7. How much automatization is possible for a PIRP in your opinion?
8. Do you think it is (will be) possible to identify the patients with a specific disease based on their communication in social media?

## Industry

1. Is there guidance on the appropriate use of social media for research (from the legal ethical perspective)? (References?)
2. Where do you see the advantages / opportunities of using such a platform?
3. Which risk or down-side in using such a platform do you see?
4. How could platform be managed and financed?

## Pharmacists or Doctors / Experts of the appraisal committee

1. Where do you see the advantages / opportunities of using such a platform?
2. Do you see any risk or downside in using such a platform?
3. In your opinion, what do you think is the added value of such a platform? *(e.g., how could a PIRP fit within the existing HTA agency process and strengthen it?)*

# Section 3 (general)

1. Do you think your country is ready to use this type of source for research?
2. Are there any other thoughts or comments which you would like to share with us?
3. Would you be interested in receiving further information throughout the project and/or in participating in the consultation process when we circulate the draft of the whitepaper?
4. Is it acceptable for you if we acknowledge your name as contributor in the whitepaper?
