# Supplementary material for: Using social media research in health technology assessment: stakeholder perspectives and scoping review
Source: Int J Technol Assess Health Care. 2023 Sep 21;39(1):e63. doi: 10.1017/S0266462323002593 (PMC11570010; doi:10.1017/S0266462323002593)
Supplement: Holtorf et al. supplementary material 2 — Holtorf et al. supplementary material [file S0266462323002593sup002.docx]

| **Thematic Analysis** |  |  |  |  |  |
| --- | --- | --- | --- | --- | --- |
|  |  |  |  |  |  |
| **Total Sample (N = 25)** | **Stakeholder Group** | | | | |
|  | **HTA Organizations (N = 5)** | **Patient & Caregiver Advocates (N = 14)** | **Academia (N = 1)** | **Industry (N = 5)** |  |
| Section 1 |  |  |  |  |  |
| 1. Do you agree with the expectations for PIRP? | Generally yes; should be considered an additional source of information, not a replacement | Generally yes; some concerns on tools & methods | Yes | Generally yes; some concerns on tools & methods |  |
| 2.  Are there additional expectations that should be met? | Unclear how to manage patients' expectations after submitting information; any information pulled would need to meet existing HTA standards for good research | Summarizing the information and give feedback to patient community; privacy & security should not be mixed; data and data analytics should be accessible for patients/public; what is the receptiveness of HTA bodies; all themes identified through social media should be validated with other methods | Transparency; Methods (Adjusting for bias) | How to handle/manage reporting potential adverse events identified; Get the information early in the HTA process; Geographic considerations; integrate information collected with all other info; how would this contribute to the sharing of knowledge; |  |
| 3.  Which additional stakeholders and should be considered? | Academia; health communication experts; family/carers for those who do not use social media; decision-makers | Regulators; pharmacovigilence experts; citizens/publics; cyber security; all stakeholders of a typical HTA; social media experts; | Regulators | Regulators; cyber security; citizens/public; policy-makers; keep the diversity of groups in mind |  |
| 4. What kind of governance would you expect? | Multi-stakeholder oversight; monitoring; may have to rely on existing platform's terms; independent platform | Government or non-profit; strict guidelines and framework; digital rights; data ownership (patients should own their own data); patient representation in any governance structure; some recommend HTAi as the group to oversee it; | National Level; Patient/Citizen Representation | Government or non-profit; patient groups engaged in oversight; quality control process needs to be in place; |  |
| 5. Who would have an interest in manipulating (e.g. hacking) / level of risk? | Patient community may have an interest in manipulating to emphasize the need of a specific group; Concern of "catfishing" scenarios; patients seeing other patient experiences different from their own | "trolls" producing fake information; anti-vaccination groups; manipulation might emerge outside of Pharma groups such as private investors; | N/A | Easy to manipulate with automation such as "chat bots"; "trolls"; |  |
| 6. Which level of transparency do you expect? | Need to be clear how the information is being used; any funding from industry must be disclosed | Mixed - some want full transparency with open access to data & full patient ownership while others want transparency with limits on who can access the data | Multiple levels expected; technical transparency; results transparency; process transparency; Do not give full transparency on each level to reduce exposure to risk of abuse | Multiple levels expected; primarily on process/methods and how results are shared |  |
| 7. Do you feel a PIRP could be a credible source for identifying unmet patient needs? | Could provide value but a long way from being "credible"; the credibility could be viewed in an appropriate context; potential use in the beginning of an assessment | Unanimously "Yes" with many including several caveats (e.g., use with other sources of data, an additional source of data but not a replacement, concerns of credibility) | Yes; Already happening | Generally yes; some concerns on data validation and methods |  |
| 8. Are you aware of any example in which social media data was used to generate patient-based evidence? | Patient groups currently gather social media information and included in HTA submission; | Evidera presented research on this at ISPOR; PatientsLikeMe; AFIP; patient groups use different forms of social media regularly but not necessarily for HTA; | Pilot projects with US FDA; Ovarian cancer on Twitter; Smoking cessation on Twitter; | COVID-19 social media listening project; internal examples under "market research" teams |  |
| Section 3 |  |  |  |  |  |
| 1. Do you think your country is ready to use this type of source for research? | Mixed | Mixed | Yes but different levels of acceptability | Mixed |  |
| 2. Are there any other thoughts which you would like to share with us? | Addressing the credibility of the information and transparency of use are critical | This is a very complex subject; important to consider multiple social media platforms; | N/A | Why 'social listening' as opposed to other methods (e.g., surveys)?; HTA bodies often have limited resources, social media platforms may offer a way to capture more information at a lower cost; |  |
