# Supplementary material for: Using social media research in health technology assessment: stakeholder perspectives and scoping review
Source: Int J Technol Assess Health Care. 2023 Sep 21;39(1):e63. doi: 10.1017/S0266462323002593 (PMC11570010; doi:10.1017/S0266462323002593)
Supplement: Holtorf et al. supplementary material 3 — Holtorf et al. supplementary material [file S0266462323002593sup003.docx]

| **Authors, Year, Source** | **Full Text Link** | **Type of Analysis** | **Type of Study** | **Media Database / Data Source** |
| --- | --- | --- | --- | --- |
| Jacques L., Carpenter E., Valley T., Alvarez B., Higgins J., 2021, American journal of obstetrics and gynecology | <http://dx.doi.org/10.1016/j.ajog.2021.05.011> | Social media data analysis | exploratory study | used Python5 to web-scrape the 250 most recent posts from United States. |
| Bour C., Ahne A., Schmitz S., Perchoux C., Dessenne C., Fagherazzi G., 2021, Journal of Medical Internet Research 23:5 Article e25736. | <http://dx.doi.org/10.2196/25736> | Machine learning or data mining techniques | Scoping review |  |
| Smith D.J., Mac V.V.T., Hertzberg V.S., 2021, Journal of nursing scholarship 53:3 (343-350). | <http://dx.doi.org/10.1111/jnu.12654> | Sentiment analysis | content analysis | twitter |
| Burton C.S., Gonzalez G., Vaculik K., Khalil C., Zektser Y., Arnold C., Almario C.V., Spiegel B.M.R., Anger J.T., 2021, Urology 150 (139-145). | <http://dx.doi.org/10.1016/j.urology.2020.06.056> | Digital analysis and social media data mining | digital analysis | anonymous online posts on social media sites collected by a social media data mining service. |
| Ehret C., Young C., Ellefson C.J., Aase L.A., Jatoi A., 2021, The American journal of hospice & palliative care . | <http://dx.doi.org/10.1177/10499091211006923> | Social media data analysis | review article | [80,000-member medical social media platform, Mayo Clinic](https://connect.mayoclinic.org/) |
| Teoh J.Y., 2021, Scandinavian journal of occupational therapy (1-11). | <http://dx.doi.org/10.1080/11038128.2021.1895307> | Thematical analysis | review article | Facebook |
| Warner E.L., Kirchhoff A.C., Wilson A., Cloyes K.G., Sun Y., Waters A.R., Nelson T., Ellington L., 2021, Journal of cancer survivorship : research and practice . | <http://dx.doi.org/10.1007/s11764-021-01004-y> | Social media data analysis | cross-sectional retrospective mixed-methods study |  |
| Fazekas B., Megaw B., Eade D., Kronfeld N., 2021, Epilepsy and Behavior 116 Article Number: 107729. | <http://dx.doi.org/10.1016/j.yebeh.2020.107729> | NLP and qualitative in-depth and contextual analysis | qualitative netnographic study | Twitter, forums. |
| Franchina V., Franchina T., Ricciardi G., Adamo V., 2021, Journal of Thoracic Oncology 16:3 Supplement (S469). | <http://dx.doi.org/10.1016/j.jtho.2021.01.809> | Data analysis | review article | Facebook |
| Khanbhai M., Anyadi P., Symons J., Flott K., Darzi A., Mayer E., 2021, BMJ health & care informatics 28:1. | <http://dx.doi.org/10.1136/bmjhci-2020-100262> | NLP & Machine learning | Systematic review | free-text patient feedback |
| Fodeh S.J., Al-Garadi M., Elsankary O., Perrone J., Becker W., Sarker A., 2021, Computers in Biology and Medicine 129 Article No: 104132. | <http://dx.doi.org/10.1016/j.compbiomed.2020.104132> | NLP & Machine learning | review article | Twitter |
| Carvalho E.S.S., doVale P.R.L.F., Pinto K.A., Ferreira S.L., 2021, Revista brasileira de enfermagem 74 Suppl 1 (e 0581). | <http://dx.doi.org/10.1590/0034-7167-2020-0581> | Thematic analysis | exploratory study | YouTube |
| Summa Linguae, 2021, Summa Linguae Technologies | <https://summalinguae.com/localization/social-listening-medical-insights/> | SML data analysis methods | Commentary |  |
| Karafillakis E, Martin S, Simas C, Olsson K, Takacs J, Dada S, et al. , 2021, JMIR Public Health and Surveillance | <https://publichealth.jmir.org/2021/2/e17149/> | Social media tools | Systematic review | across all socia media platforms (Twitter, YouTube, Facebook) |
| Research Partnership / Inizio Advisory,, 2021, University of Manchester, | <https://www.researchpartnership.com/resources/case-study/how-we-uncovered-unmet-needs-using-social-media-listening> | SML data analysis | Case study |  |
| UK Research & Innovation, 2021, University of Manchester, MR/S004025/1 | <https://gtr.ukri.org/projects?ref=MR%2FS004025%2F1> | NLP | Active research | twitter, blogs and online discussion forums |
| Chris Miller, 2021, Finances Online | https://financesonline.com/social-media-data-mining-techniques/#:~:text=Social%20Media%20Data%20Mining%20Techniques%20You%20Should%20Know,media%20data%20mining%20software | Social Media Data Mining Techniques | Commentary |  |
| (anonymous), 2021, Java T Point | <https://www.javatpoint.com/social-media-data-mining-methods> | Social Media Data Mining Methods | Commentary |  |
| Michal Podhoranyi, 2021, Springer Link | <https://link.springer.com/article/10.1007/s12145-021-00601-w> | Social media data processing and analytics | Case study | Twitter |
| Nikita Duggal, 2021, Simplilearn | <https://www.simplilearn.com/what-is-data-processing-article> | Types of data processing | Commentary |  |
| Hanh Truong, 2021, Any Connector | <https://anyconnector.com/data-transformation/what-is-data-wrangling.html> | Data wrangling steps | Commentary |  |
| Oleksandr Bushkovskyi, 2021, The App Solutions | <https://theappsolutions.com/blog/development/data-wrangling-guide-to-data-preparation/> | Data wrangling steps | blog |  |
| Express Analytics, 2021, Express Analytics | <https://expressanalytics.com/blog/what-is-data-wrangling-what-are-the-steps-in-data-wrangling/> | Data wrangling steps | Commentary |  |
| Jane Thompson, 2021, NETBASEQUID | <https://netbasequid.com/blog/free-social-media-analytics-tools/> | Social media analytics tools | Commentary |  |
| Halhol S., Raluy-Callado M., Oguz M., Booth A., 2020, Value in Health 23 Supplement 2 (S474). | <http://dx.doi.org/10.1016/j.jval.2020.08.430> | Thematic analysis | N.A. |  |
| Faust G., Booth A., Merinopoulou E., Halhol S., Tosar H., Nawaz A., Szlachetka M., Chiu G., 2020, Annals of Oncology 31 Supplement 4 (S763). | <http://dx.doi.org/10.1016/j.annonc.2020.08.1264> | Machine learning | Retrospective study | Health-related social media |
| Denham A.M., Baker A.L., Spratt N.J., Wynne O., Hunt S.A., Bonevski B., Kumar R., 2020, Health informatics journal 26:3 (1599-1616). | <http://dx.doi.org/10.1177/1460458219873538> | YouTube video data analysis | online YouTube search engine. | videos in YouTube |
| Sullivan T.F., 2020, Infection Control and Hospital Epidemiology 41:9 (1096-1098). | <http://dx.doi.org/10.1017/ice.2020.265> | Social media data analysis | Retrospective study | Twitter in the United States via the Twitter developer platform. |
| El-Awaisi A., O'Carroll V., Koraysh S., Koummich S., Huber M., 2020, Journal of interprofessional care 34:5 (622-632). | <http://dx.doi.org/10.1080/13561820.2020.1819779> | Social media data analysis | A cross-sectional retrospective review | Facebook®, Twitter®, and LinkedIn®. |
| Haruta J., Tsugawa S., Ogura K., 2020, Family medicine and community health 8:4. | <http://dx.doi.org/10.1136/fmch-2020-000396> | Social network analysis | Social network analysis | Social network analysis based information sharing clinical network data |
| Jiang T., Osadchiy V., Mills J.N., Eleswarapu S.V., 2020, Urology 142 (133-140). | <http://dx.doi.org/10.1016/j.urology.2020.04.100> | NLP | quantitative natural language processing (NLP) and qualitative annotation of content | Reddit community |
| Yoo D.W., Birnbaum M.L., van Meter A.R., Ali A.F., Arenare E., Abowd G.D., de Choudhury M., 2020, JMIR Mental Health 7:8 Article Number: e16969. | <http://dx.doi.org/10.2196/16969> | Social media data analysis | long-term co-design activity | patients’ social media data. |
| Wilson S., Mogan S., Kaur K., 2020, International journal of nursing practice 26:4 (e12833). | <http://dx.doi.org/10.1111/ijn.12833> | Thematic analysis | Thematic analysis | Facebook group |
| Martinelli F., Garbi A., 2020, International Journal of Gynecological Cancer 30:8 (1101-1107). | <http://dx.doi.org/10.1136/ijgc-2020-001585> | Social media data analysis | A anonymous survey | visualizations, engagement |
| Currin-McCulloch J., Stanton A., Boyd R., Neaves M., Jones B., 2020, Psychology & health (1-18). | <http://dx.doi.org/10.1080/08870446.2020.1792903> | Thematic analysis | Qualitative analysis | online focus groups |
| Yousuf H., Corbin J., Sweep G., Hofstra M., Scherder E., Van Gorp E., Zwetsloot P.P., Zhao J., Van Rossum B., Jiang T., Lindemans J.-W., Narula J., Hofstra L., 2020, JAMA Network Open 3:7 Article Number: e2014323. | <http://dx.doi.org/10.1001/jamanetworkopen.2020.14323> | Social media data analysis | Cross-sectional analysis of survey | Survey distributed by a large national newspaper and a popular social influencer |
| Lee Y.J., Park A., Roberge M., Donovan H., 2020, Cancer nursing . | <http://dx.doi.org/10.1097/NCC.0000000000000860> | Social media data analysis | topic modeling (ie, latent Dirichlet allocation [LDA]) | patient/caregiver posts from an online forum. |
| Tan Y., Teng Z., Qiu Y., Tang H., Xiang H., Chen J., 2020, JMIR mHealth and uHealth 8:7 (e16215). | <http://dx.doi.org/10.2196/16215> | Social media data analysis | A web-based survey | questionnaires |
| Gao S., He L., Chen Y., Li D., Lai K., 2020, Journal of Medical Internet Research 22:7 Article Number: e16649. | <http://dx.doi.org/10.2196/16649> | Content analysis | Analysis | Sina Weibo |
| Hui C.Y., Walton R., McKinstry B., Pinnock H., 2020, Health informatics journal 26:2 (862-879). | <http://dx.doi.org/10.1177/1460458219853381> | Thematic analysis | Analysis | Usage data. Questionnaire data. Qualitative interviews. |
| Osadchiy V., Mills J., Eleswarapu S., 2020, Journal of Urology 203 Supplement 4 (e666). | <https://pubmed.ncbi.nlm.nih.gov/33026354/> | NLP | Analysis | Reddit community |
| Oser T.K., Oser S.M., Parascando J.A., Hessler-Jones D., Sciamanna C.N., Sparling K., Nease D., Litchman M.L., 2020, Current Diabetes Reports 20:3 Article Number: 10. | http://dx.doi.org/10.1007/s11892-020-1294-3 | Social media data analysis | Review Article | A PubMed search of the terms “diabetes” and “social media” |
| Sarker A., Deroos A., Perrone J., 2020, Journal of the American Medical Informatics Association 27:2 (315-329). | <http://dx.doi.org/10.1093/jamia/ocz162> | Social media data | Review Article | Medline and Embase, Scopus, Web of Science, and Google Scholar |
| Gijsen V., Maddux M., Lavertu A., Gonzalez-Hernandez G., Ram N., Reeves B., Robinson T., Ziesenitz V., Shakhnovich V., Altman R., 2020, Clinical and Translational Science 13:1 (26-30). | <http://dx.doi.org/10.1111/cts.12687> | Social media data analysis | Commentary | N. A. |
| Green B.M., van Horn K.T., Gupte K., Evans M., Hayes S., Bhowmick A., 2020, Journal of Medical Internet Research 22:7 Article No.: e17338. | <http://dx.doi.org/10.2196/17338> | Content analysis | Analysis | Facebook’s analytic tool, Facebook Insights. |
| Correia RB, Wood IB, Bollen J, Rocha LM. , 2020, Annu Rev Biomed Data Sci [Internet]. ;3:433–58. | <https://www.ncbi.nlm.nih.gov/pmc/articles/PMC7299233/> | Social media data analysis | Review Article (Sentiment analysis tools) | N. A. |
| George Regkas, 2020, Towards Data Science | <https://towardsdatascience.com/leveraging-on-nlp-to-gain-insights-in-social-media-news-broadcasting-ca89752ef638> | NLP | Commentary | N. A. |
| (anonymous), 2022, Saras | <https://sarasanalytics.com/blog/data-wrangling> | Data wrangling steps & tools | Commentary | N. A. |
| Nick Monaco, Daniel Arnaudo, 2020, NDI.ORG | <https://www.ndi.org/sites/default/files/NDI_Social%20Media%20Monitoring%20Guide%20ADJUSTED%20COVER.pdf> | Guidance on Social Media Monitoring and Analysis Techniques, Tools and Methodologies | Guidance | N. A. |
| (anonymous), 2020, Keyhole | <https://keyhole.co/blog/list-of-the-top-25-social-media-analytics-tools/> | Social Media Analytics Tools | Social Media Analytics Tools | N. A. |
| Tobias Geisler Mesevage, 2020, MonkeyLearn | <https://monkeylearn.com/blog/social-media-sentiment-analysis-tools/> | Sentiment Analysis Tools | Sentiment Analysis Tools to Monitor Social Media Data | N. A. |
| Marta, 2020, BRAND24 | <https://brand24.com/blog/guide-to-social-media-analysis/> | Social media analysis | A guidance to social media analysis | N. A. |
| Josh Miramant, 2020, UNITE.AI | <https://www.unite.ai/6-steps-to-get-insights-from-social-media-with-natural-language-processing/> | Sentiment Analysis and NLP | Sentiment Analysis and the NLP | N. A. |
| María N. Moreno-García, 2020, MDPI | <https://www.mdpi.com/2078-2489/11/12/578/htm> | Information retrieval and social media mining | Editorial | N. A. |
| Jiawei Li; Qing Xu; Raphael Cuomo; Vidya Purushothaman; Tim Mackey, 2020, JMIR Public Health Surveill ;6(2):e18700 | <https://publichealth.jmir.org/2020/2/e18700/> | Social media infoveillance study | quantitative analysis, | Chinese-language messages from Wuhan on Weibo. |
| Cuihua Shen; Anfan Chen; Chen Luo; Jingwen Zhang; Bo Feng; Wang Liao, 2020, J Med Internet Res ;22(5):e19421 | <https://www.jmir.org/2020/5/e19421/> | Social media infoveillance study | Analysis | Weibo user pool of 250 million people |
| Li Y., Atkinson T., Bochner B.H., Rapkin B.D., 2019, Quality of Life Research 29:SUPPL 1 (S86). | <http://dx.doi.org/10.1007/s11136-020-02626-y> | NLP & Sentiment analysis | Conference Abstract |  |
| Langford A., Loeb S., 2019, Journal of medical Internet research 21:5 (e13512). | <http://dx.doi.org/10.2196/13512> | Descriptive statistics and multivariable logistic regression | Nationally representative cross-sectional analysis | Health Information National Trends Survey |
| Dong Y., Zhou X., Lin Y., Pan Q., Wang Y., 2019, PLoS ONE 14:2 Article Number: e0213066. | <http://dx.doi.org/10.1371/journal.pone.0213066> | Word clouds and coding schemes | Analysis | Baidu HIV-related bar, TB-related bar |
| Jiménez-Zafra S.M., Martín-Valdivia M.T., Molina-González M.D., Ureña-López L.A., 2019, Artificial Intelligence in Medicine 93 (50-57). | <http://dx.doi.org/10.1016/j.artmed.2018.03.007> | Sentiment analysis | supervised learning and lexicon-based sentiment analysis | two forums. |
| Stokes Y., Vandyk A., Squires J., Jacob J.-D., Gifford W., 2019, Western journal of nursing research 41:1 (96-110). | <http://dx.doi.org/10.1177/0193945917740706> | Social media data | cross-sectional analysis | survey in Facebook, LinkedIn |
| Pappa D, Stergioulas LK. , 2019, Int J Data Sci Anal. ;8(2):113–35. | https://link.springer.com/article/10.1007/s41060-019-00175-3 | Social media data analysis methods | Review Article | Multiple |
| Cook N, Mullins A, Gautam R, Medi S, Prince C, Tyagi N, et al. , 2019, Ophthalmol Ther. ;8(3):407–20. | <https://www.ncbi.nlm.nih.gov/pmc/articles/PMC6692792/> | NLP | cross-sectional analysis | Multiple |
| Carrillo-de-Albornoz J., Vidal J.R., Plaza L., 2018, PLoS ONE 13:11 Article Number: e0207996. | http://dx.doi.org/10.1371/journal.pone.0207996 | Sentiment analysis | cross-sectional analysis | Propietary site called "MedHelp" |
| Hswen Y., Sewalk K.C., Alsentzer E., Tuli G., Brownstein J.S., Hawkins J.B., 2018, Social Science and Medicine 215 (92-97). | http://dx.doi.org/10.1016/j.socscimed.2018.08.031 | Social media data analysis | cross-sectional analysis | Twitter |
| De Silva D., Ranasinghe W., Bandaragoda T., Adikari A., Mills N., Iddamalgoda L., Alahakoon D., Lawrentschuk N., Persad R., Osipov E., Gray R., Bolton D., 2018, PLoS ONE 13:10 Article Number: e0205855. | <http://dx.doi.org/10.1371/journal.pone.0205855> | NLP & Machine learning | Cohort | "online support groups from 10 high volume active groups" |
| Koltai K., Walsh C., Jones B., Berkelaar B.L., 2018, Journal of Adolescent and Young Adult Oncology 7:2 (181-186). | http://dx.doi.org/10.1089/jayao.2017.0058 | Social network analysis | Need full text |  |
| Arigo D, Pagoto S, Carter-Harris L, Lillie SE, Nebeker C. , 2018, Digit Health [Internet]. 4. | https://www.ncbi.nlm.nih.gov/pmc/articles/PMC6016568/ | Social media data analysis methods | Commentary | NA |
| IDEYA, 2018, Ideya Market Report 9th Edition | <http://ideya.eu.com/images/SMMTools%20Excerpts%202018%20Final.pdf> | Social media monitoring tools | Guidance / Report | Multiple |
| Vosburg S.K., Haynes C., Besharat A., Green J.L., 2017, Pharmacoepidemiology and Drug Safety 26:9 (1044-1052). Date of Publication: 1 Sep | http://dx.doi.org/10.1002/pds.4248 | Social media data | cross-sectional analysis | Multiple |
| Carroll C.L., Bruno K., Ramachandran P., 2017, Chest 152:2 (402-409). | <http://dx.doi.org/10.1016/j.chest.2017.03.003> | Symplur analytics | Cohort | Twitter |
| Hammer M.J., 2017, Oncology nursing forum 44:4 (410-412). | http://dx.doi.org/10.1188/17.ONF.410-412 | Ethical Considerations | Commentary | NA |
| Hindman F.M., Bukowitz A.E., Reed B.N., Mattingly T.J., 2017, Journal of the American Pharmacists Association 57:3 (318-325). | http://dx.doi.org/10.1016/j.japh.2017.01.009 | Social media data analysis | cross-sectional analysis | Instagram |
| Anu Sharma, Dr. M.K Sharma & Dr. R.K Dwivedi, 2017, Advances in Computational Sciences and Technology Vol. 10/5 pp. 1337-1354 | <https://www.ripublication.com/acst17/acstv10n5_61.pdf> | Data Mining Techniques | Review Article | Multiple |
| (anonymous), , Crimson Hexagon , Report | <https://www.upa.it/static/upload/the/the-fundamentals-of-social-media-analytics.pdf> | Social Media Analytics | Guidance / Report | Multiple |
