# Supplementary material for: Using social media research in health technology assessment: stakeholder perspectives and scoping review
Source: Int J Technol Assess Health Care. 2023 Sep 21;39(1):e63. doi: 10.1017/S0266462323002593 (PMC11570010; doi:10.1017/S0266462323002593)
Supplement: Holtorf et al. supplementary material 4 — Holtorf et al. supplementary material [file S0266462323002593sup004.docx]

# Literature Review: Case Studies for the Use of Social Media Research in Health Policy or Decision Making

| **Authors, Year, Source** | **URL** | **Region / Country** | **Policy Level addressed** | **Healthcare area** | **Social media type** | **Policy Intention** | **(Potential) Impact** |
| --- | --- | --- | --- | --- | --- | --- | --- |
| Majmundar, Anuja, Jon-Patrick Allem, Tess Boley Cruz, Jennifer B Unger, and Mary Ann Pentz. 2021. Nicotine & Tobacco Research, no. ntab085 (May). | <http://dx.doi.org/10.1093/ntr/ntab085> | USA | Public Health / FDA | tobacco and marijuana-related attitudes and behaviors | Twitter | Findings from this study should inform the FDA’s product review and standards procedures for tobacco products that can be used with marijuana. | this study offers inputs for designing comprehensive FDA regulations including regulating product features associated with appeal, improving enforcement to curb sales of illicit products, and informing the FDA’s product review and standards procedures for tobacco products that can be used with marijuana. |
| Lyu, Joanne Chen, and Garving K. Luli. 2021. Journal of Medical Internet Research 23 (2): e25108. | http://dx.doi.org/10.2196/25108 | USA | Public Health / CDC | Perceptions of CDC in the public COVID-19 related discussions | Twitter | This study aims to identify the topics and their overarching themes emerging from the public COVID-19-related discussion about the CDC on Twitter and to further provide insight into public's concerns, focus of attention, perception of the CDC's current performance, and expectations from the CDC. | 16 main topics were identified with four overarching themes: knowing the virus and the situation, policy and government actions, response guidelines, and general opinion about credibility. Social media communication can provide valuable information on public opinion. Quickly and efficiently identifying key topics within the public discussion could help public health agencies improve the communication with the public. |
| Jung, Jinho, Courtney Bir, Nicole Olynk Widmar, Peter Sayal. 2021. Journal of Food Protection 84 (7): 1150–59. | (1)<http://dx.doi.org/10.4315/JFP-20-383>  (Full text not available) | USA | Food safety / FDA, Food Safety and Inspection Service (FSIS), CDC | food safety–related incidents and recalls; public perceptions | Not specified | Social media analytics can provide insights into public awareness of food safety–related incidents. This study included social listening data to analyze how the public, in social and online media spaces, responds to, interacts with, and references food safety recalls and/or initial announcements of foodborne illness outbreaks as reported by the Centers for Disease Control and Prevention (CDC). | Analysis results suggest that mentions quantified in the social and online media searches moved closer in step with the CDC's initial reports of foodborne illness outbreaks than did FDA and FSIS recall announcements. Recall announcements by the FDA and FSIS may not induce changes in consumers' behavior, whereas initial illness reports by the CDC may. Online media analytics provide policy makers with information to guide effective food risk communication; initial CDC reports drive immediate attention more than do FDA and FSIS recalls. |
| Laestadius, Linnea I, Megan M Wahl, Julia Vassey, and Young Ik Cho. 2020. Nicotine & Tobacco Research 22 (10): 1823–30. | http://dx.doi.org/10.1093/NTR/NTAA092 | USA | FDA policy implementation | Tobacco use | Instagram | This study examines use of warnings on promotional Instagram posts before and after provisions took effect on August 10, 2018.  Checking, how much the legal requirement for safety warning is implemented in social media. | In the prewarning period, nicotine warning statements were absent on all posts. Following August 10, 2018, FDA compliant warnings were present on 13.6% of posts (global) and 36.4% if based in the USA. The share of posts made by US Instagram users decreased by 11%, but total post volume continued to grow. |
| Luisi, Monique L. R. 2021. Vaccine 39 (2): 303–8. | http://dx.doi.org/10.1016/j.vaccine.2020.11.065 | USA | FDA decision – perceived risk / Academic | HPV vaccination | Facebook | Social amplification of risk framework concepts with HPV vaccination were analyzed to measure their relationships with post characteristics, engagement, and to see how those variables changed over time. | Nearly four out of every ten Facebook posts about the HPV vaccine contained messages that amplified the risk of HPV vaccine and the data suggest that these posts had momentum over time. Research must continue to address the perception of vaccine safety where the vaccine is perceived as the health threat, with deep research into online communities to discover the perceived ripples and impacts. |
| Fadaee, Negin, Desmond Huynh, and Shirin Towfigh. 2020. The American Surgeon 86 (10): 1351–57. | http://dx.doi.org/10.1177/0003134820964459 | USA | FDA regulation / impact of social media advertisement by lawyers in FDA regulation | Surgical hernia mesh / public perceptions | Twitter and Facebook | By 2016, social media discussion against the use of transvaginal mesh influenced changes in Food and Drug Administration (FDA) regulations. Authors compare the trend of social media discussion of hernia and transvaginal mesh. | On Facebook, 16 public groups with 14 526 members expressed negative sentiments in 95% of their 750 daily posts. Of the 1.1 million tweets on Twitter, those about hernia mesh were more negative (36.5%) than those about pelvic/vaginal mesh (29.2%). Three of the 5 top tweeters about hernia mesh and pelvic/vaginal mesh were linked to law firms involved in mesh-based lawsuits. The negative sentiments and steering of social media discussion by lawyers may directly affect surgical care. As surgeons, we may adapt our informed consent to acknowledge our patients' apprehensions about mesh. We may also be more involved in social media discussions ourselves. |
| Lee, Christine, Christopher St Clair, CDR Christine Merenda, CAPT Richardae Araojo, Sara Ray, Derrick Beasley, and RADM Denise Hinton. 2020. Research in Social and Administrative Pharmacy 16 (7): 967–73. | http://dx.doi.org/10.1016/j.sapharm.2019.10.009 | USA | Pilot study by FDA; Patient experience data to inform FDA understanding of patient experiences | Study 1: Opioid Use Disorder (OUD).  Study 2: Pulmonary Arterial Hypertension (PAH) | 1. public docket^[[1]](#footnote-1)^  comments and social media data.  2. Comments on FDA meeting transcripts | Two observational studies to show how observational and social science methodology can be applied to new data sources (social media) as well as FDA archival data sources. | Triangulating multiple data sources increases the comprehensive confidence in narrative information. Using these varied data sources provided access to and information from people with rare and/or stigmatized conditions, those living in geographically disparate areas of the country, and those with disabilities that make it difficult for them to participate in research. |
| Patalano, Francesco, Florian S. Gutzwiller, Bhavik Shah, Chitresh Kumari, and Nigel S. Cook. 2020. Advances in Therapy 37 (1): 17–26. | http://dx.doi.org/10.1007/s12325-019-01134-x | global | Pharma industry🡪 Patient Centric Clinical Research | COPD | publicly accessible sources  without accessing password-protected information | Structured Patient Insight to Drive the PRO Strategy in COPD | Social media listening (SML) study, online bulletin board (OBB) exercise, and design of an online patient preference study (PPS). The initial online studies (SML and OBB) revealed that, besides dyspnoea and exacerbations, patients perceive cough and mucus production as equally important aspects of disease management for COPD. |
| Golder, Su, Karen Smith, Karen O’Connor, Robert Gross, Sean Hennessy, and Graciela Gonzalez-Hernandez. 2021. Drug Safety 44 (2): 167–79. | http://dx.doi.org/10.1007/s40264-020-00998-1 | USA (UK) | Academic research; concept testing for pharmacovigilance | Adverse event identification; Statins | Twitter | to assess the consistency of adverse event data of statin medications from social media as  compared with other sources | Combining the data from multiple sources, albeit challenging, may provide a broader safety profile of any medication. |
| Stekelenborg, John van, Johan Ellenius, Simon Maskell, Tomas Bergvall, Ola Caster, Nabarun Dasgupta, Juergen Dietrich, et al. 2019. Drug Safety 42 (12): 1393–1407. | http://dx.doi.org/10.1007/s40264-019-00858-7 | Europe | WEB-RADR = IMI-Project | Adverse event identification | Facebook, Twitter, Reddit and online patient communities such as Inspire | The WEB-RADR project has developed a collaborative English language workspace for visualising and analysing social media data for a number of medicinal products. | - General social media, as exemplified by sample data from Facebook and Twitter, are not recommended for broad statistical signal detection. - Social media channels may provide a useful adjunct to pharmacovigilance activities in specific niche areas such as exposure during pregnancy and abuse/misuse of medicines. - Future enhancement of adverse event recognition algorithms may broaden the scope and utility of social media over time. |
| Timimi, Farris, Sara Ray, Erik Jones, Lee Aase, and Kathleen Hoffman. 2019. Journal of Medical Internet Research 21 (11): e14809. | http://dx.doi.org/10.2196/14809 | USA | Research to inform FDA; (promotion for Inspire platform) | Adverse event identification / Statins | user-generated content on Inspire | to apply both natural language processing (NLP) technology and hands-on linguistic analysis to a set of online posts from known statin users to (1) identify any underlying crossover between the use of statins and impairment of memory or cognition and (2) obtain patient lexicon in their descriptions of experiences with statin medications and memory changes. | By looking at posts from statin users about memory, four key themes were found and described in detail in the data: memory loss, aphasia, cognitive impairment, and emotional change. For side effects such as those on memory and cognition, where self-reporting may be unreliable, these methods can provide another avenue to inform patients, providers, and the Food and Drug Administration. |
| Booth, Alison, Timothy Bell, Sonia Halhol, Shiyu Pan, Verna Welch, Evie Merinopoulou, Dimitra Lambrelli, and Andrew Cox. 2019. Journal of Medical Internet Research 21 (11): e14285. | http://dx.doi.org/10.2196/14285 | USA / UK / global | Pharma industry🡪 Patient Centric Clinical Research | Acute Myeloid Leukemia or Myelodysplastic Syndrome | publicly available discussions on 3 large AML- or MDS–specific sites | to use disease-specific social media posts by patients with AML or MDS who are ineligible for intensive chemotherapy and their caregivers to capture factors they feel are most important, and to provide current evidence to inform and characterize these perspectives. | disease-specific social media communication highlighted experiences in the current treatment of AML and MDS, including information gaps, patient/caregiver uncertainty, and the importance of understanding patients’/caregivers’ goals and opinions |
| Nikfarjam, Azadeh, Julia D Ransohoff, Alison Callahan, Vladimir Polony, and Nigam H Shah. 2019. JAMIA Open 2 (3): 301–5. | http://dx.doi.org/10.1093/jamiaopen/ooz025 | USA | FDA / Proof of concept for off-label drug use | Off-label drug use identification | Inspire health forums (www.inspire.com) | To investigate using patient posts in social media as a resource to profile off-label prescriptions of  cancer drugs. | identified 279 frequently discussed and therefore highly associated drug-disease pairs from Inspire posts. Of these, 96 are FDA approved, 9 are known off-label uses, and 174 do not have records of known usage (potentially novel off-label uses). We achieved a mean average precision of 74.9% in identifying drug-disease pairs with a true indication association from patient posts and found consistent evidence in medical claims records. We achieved a recall of 69.2% in identifying known off-label drug uses (based on Wolters-Kluwer Medi-span) from patient posts.  🡪 proof-of-principle detection of off-label drug use from patient-generated content in social media using text mining methods |
| Cook, Nigel S., Julie Cave, and Anke-Peggy Holtorf. 2019. Frontiers in Medicine 6 (Art. 82): 17. | http://dx.doi.org/10.3389/fmed.2019.00082 | global | Pharma industry🡪 Patient Centric Clinical Research | Drye Eye Disease, NASH | Not defined; publicly accessible social media posts | To better address unmet patient needs in clinical development | The authors advocate that all healthcare players should actively contribute to aligning on best practices concerning choice of methodologies and engage in multi-stakeholder dialog along the entire product development chain, to realize health technologies that best meet the needs of patients. |
| *(anonymous),* 2019, *Qual Life Res* **28** (Suppl 1), 1–190 | <http://dx.doi.org/10.1007/s11136-019-02257-y> | USA | FDA | Diabetes | Twitter | The agency’s regulatory mission relies on sourcing new data and methodologies to increase its understanding of patients’ perspectives. The FDA uses patient experience data to inform:  • Clinical trial design  • Trial end point development and selection  • Regulatory issues, including benefit-risk assessments  To meet patients’ needs, the FDA strives to engage patient stakeholders throughout the life cycle of a medical product. | Although the enhanced patient insight is encouraging (gained from social media posts), social media data must be combined with other sources of data, including FDA archives, patient-focused drug development data, public docket comments, advisory council transcripts, focus groups, and listening sessions. |
| Convertino, Irma, Sara Ferraro, Corrado Blandizzi, and Marco Tuccori. 2018. Expert Opinion on Drug Safety 17 (11): 1081–93. | http://dx.doi.org/10.1080/14740338.2018.1531847 | Global | Academic / Safety / Pharmacovigilance Methods | Adverse event identification (negative; not recommended) | general | This systematic review is aimed at evaluating the usefulness and quality of proto-signals by social media listening. | … poorer information quality as compared with spontaneous reporting databases. This feature allows rarely the evaluation of causal relationships. Proto-signals identified by social media listening had the potential of anticipating pre-specified known signals in only six studies. Moreover, the personal perception of patients reported in social media could be used to implement effective risk communication strategies. However, signal detection in social media cannot be currently recommended for routine pharmacovigilance, due to logistic and technical issues. |
| Chen, Xiaoyi, Carole Faviez, Stéphane Schuck, Agnès Lillo-Le-Louët, Nathalie Texier, Badisse Dahamna, Charles | http://dx.doi.org/10.3389/fphar.2018.00541 | France | Academic / Testing of method | Adverse event identification / Ritalin (methylphenidate) | five popular and open French forums: www.atoute.org, www.doctissimo.fr, www.e-sante.fr, www.onmeda.fr (previously www.aufeminin.com) and sante-medecine.journaldesfemmes.com | To test text mining methods based on named entity recognition and relation extraction in the corpus, followed by signal detection using proportional reporting ratio (PRR). | Named entity recognition combined with signal detection and topic modeling have demonstrated their complementarity in mining social media data. An in-depth analysis focused on methylphenidate showed that this approach was able to detect potential signals and to provide better understanding of patients' behaviors regarding drugs, including misuse. |
| Beasly, D, C Lee, C Merenda, T Toebe, and J Boudreaux. 2018. https://doi.org/10.1016/j.japh.2018.04.004. | http://dx.doi.org/10.1016/j.japh.2018.04.004 | USA | FDA | Diabetes | Facebook; unstructured FDA data for advisory committees and public workshops that mentioned “diabetes” and/or “insulin,” capturing perspectives from patients, industry, advocate groups, and health practitioners / researchers. | The study objectives are to use qualitative research methods to characterize themes related to FDA public workshops and advisory committees focused on diabetes; describe patterns that emerge between traditional FDA routes of data collection from the public (advisory committees and public docket comments) and diabetes communication on Facebook; and offer insight into how unstructured data sources (both FDA and social media) can be systematically used by the agency to further support its mission to protect and improve public health. | Findings show that valuable yet different information can be found from FDA unstructured data and social media data. Additionally, this research sheds light on FDA’s strategic priority to understand the patient’s voice, including CDER’s commitment under the fifth authorization of the Prescription Drug User Fee Act (PDUFA V) that aims to more systematically gather patients’ perspectives on their condition and available treatments. |
| Rausch, Center for Drug Evaluation and. 2018. FDA. | https://www.fda.gov/drugs/news-events-human-drugs/cder-conversation-monitoring-social-media-better-understand-drug-use-trends | USA | FDA / CDER | Pharmacovigilance / general | Twitter, Facebook, and Instagram, but it also includes forums, blogs, discussion groups, news sites, and other nontraditional sites | Social media derrived data can help us identify the areas we want to explore in greater depth through more detailed and rigorous primary data collection efforts. When we monitor social media platforms, we are trying to get the broadest swath of information that we can about how people are talking about a particular issue.  For our broader social science research projects, we often conduct a social media analysis as part of our environmental scanning process, which might include reviews of peer-reviewed articles in the literature, and of non-social media websites and campaigns. We also conduct social media research on narrower topics, such as on a specific opioid, or for specific time periods, such as over a five-year period or the last 30 days. These sources give us a basic framework of information. |  |
| Seifert, Harry A., Raleigh E. Malik, Mondira Bhattacharya, Kevin R. Campbell, Sally Okun, Carrie Pierce, Jeffrey | http://dx.doi.org/10.1016/j.ahj.2017.08.021 | USA, UK | FDA and UK MHRA; think tank; cosponsored by the Drug Information Association and the Cardiac Safety Research Consortium | Adverse event identification; Cardiac safety | monitoring public conversations on the Internet: Facebook, Twitter, Inspire, Reddit, Health forums, | A major goal is to determine the feasibility of bringing together safety signal detection methodologies with the advantages and broad access of social media screening—social listening—to yield earlier, actionable insights regarding patient reports of medical product adverse events (AEs) | Although social media represent a novel, global, and publicly accessible source of data, whether or not they may eventually be used as an effective tool for postmarketing safety evidence for pharmacovigilance remains unclear. The DIA-CSRC think tank/incubator discussed in this article highlighted many unknowns regarding the value and validity of social media to supplement medical product signal detection, particularly cardiac safety signal detection. |
| Mullins, A, S Medi, N Cook, B Sloesen, C Prince, S Anand, N Tyagi, and J Kommineni. 2017. Value in Health 20 (9): A807. | http://dx.doi.org/10.1016/j.jval.2017.08.2408 | Global | Pharma industry🡪 Patient Centric Clinical Research | Dry Eye Disease | Twitter, Blogs, Forums and Newswires | To understand evolving trends in social media on dry eye disease (DED) and generate valuable insights on patients’ perceptions of disease burden, diagnosis, treatment, unmet needs and quality of life (QoL). Aim: to use information in early scientific advice. | While the study suggests a need to increase awareness about DED among patients, lack of standard diagnostic tools, treatment options and specialists emerged as key unmet needs. Poor QoL is also highlighted by patients with significant impact on daily activities, work and commute. Further exploration of QoL also revealed a huge gap in patient emotional needs. Fear, anger and sadness were expressed by 20%, 18% and 30% posts respectively. Additionally, 1% of patient posts also indicated suicidal tendency. |
| Pierce, Carrie E., Khaled Bouri, Carol Pamer, Scott Proestel, Harold W. Rodriguez, Hoa Van Le, Clark C. Freifeld, et al. 2017. Drug Safety 40 (4): 317–31. | http://dx.doi.org/10.1007/s40264-016-0491-0 | USA | FDA | Adverse event identification / detection | Facebook, Twitter | Our objective was to examine whether specific product–adverse event pairs were reported via social media before being reported to the US FDA Adverse Event Reporting System (FAERS).  A retrospective analysis of public Facebook and Twitter data was conducted for 10 recent FDA postmarketing safety signals at the drug–event pair level with six negative controls | Of 10 recent postmarketing safety signals from the US FDA, public Facebook and Twitter posts showed mentions of one product–event pair before cases were reported to the traditional spontaneous report system.  Social media can be an adjunct to traditional safety reporting systems to possibly uncover postmarketing safety signals more rapidly, although considerations of noise and volume need to be further characterized. |
| Rose, Shyanika W, Catherine L Jo, Steven Binns, Melissa Buenger, Sherry Emery, and Kurt M Ribisl. 2017. Journal of Medical Internet Research 19 (2): e56. | http://dx.doi.org/10.2196/jmir.5694 | USA | Public health | Smoking prevention / communication | Twitter | to identify the content and frequency of conversations about menthol cigarettes, including themes, populations, user smoking status, other tobacco or substances, tweet characteristics, and sentiment. Aim: to inform communication about these products, particularly to subgroups who are at risk for menthol cigarette use. | Most messages are generated by smokers who have more positive sentiment toward these harmful combustible products. Misperceptions of menthol cigarettes having medicinal effects are prevalent, and positive linkage with marijuana is common. Addressing these common misconceptions and denormalizing menthol cigarette use, particularly for African American smokers, could ultimately save lives. |
|  |  |  |  |  |  |  |  |
|  |  |  |  |  |  |  |  |

**Inclusion**: 23 publications

**Countries / Regions**: Global (5), USA (16), UK (3), France (1), Europe (1),

**Policy Level / Organization**: Regulatory / MHRA /FDA (13), Public Health (3), Pharma ind / Clinical trial endpoints (4), Academic (4), Platform owner (2)

**Healthcare Area**: Tobacco / Marihuana (3), Adverse events / Pharmacovigilance (8), COPD (1), Drye Eye Disease (2), Cardiac safety (1), Ritalin (methylphenidate) (1), Diabetes (2), Acute Myeloid Leukemia or Myelodysplastic Syndrome (1), Off-label drug use (1), Statins (2), Opioid Use Disorder (OUD) (1), Pulmonary Arterial Hypertension (PAH)identification (1), Perceptions of CDC in the public COVID-19 related discussions (1), food safety–related incidents and recall related public perceptions (1), Surgical hernia mesh public perceptions (1), HPV vaccination risk perception (1)

**Social media type/data sources**: Twitter (11), Facebook (7), Patient / Health forums (6), Inspire (4), Instagram (2), Blogs (2), Newswires (2), Reddit (2), unstructured FDA data (2), public dockets (1), general / unspecified (5); specifically mentioned French open discussion forums: www.atoute.org, www.doctissimo.fr, www.e-sante.fr, www.onmeda.fr (previously www.aufeminin.com), sante-medecine.journaldesfemmes.com

**Summary of findings:**

- No concrete case where SMR has directly impacted HTA.
- However, there are examples where social media discussions have impacted health-care related decisions - even if not evidence based. Fadaee et al. describe research on anti-hernia mesh communication in social media in the USA, which was to a large degree driven by lawyer. They warned that this negative, but not evidence-based information might provoke a negative FDA decision as it has happened before with pelvic/vaginal mesh. Three of the 5 top tweeters about hernia mesh and pelvic/vaginal mesh were linked to law firms involved in mesh-based lawsuits. (2)
- There are also no examples reported, where SMR has impacted other health policy decisions.
- One of the studies included in this analysis aimed to describe patient experience for the use by FDA and the authors performed two pilot analyses in two health areas, Opioid Use Disorder (OUD) and Pulmonary Arterial Hypertension (PAH) (3).
- A lot of work has been done around using SMR for adverse event identification or detection - by the FDA, academic researchers, and the European WEB-RADR consortium (4–13). WEB-RADR come to the conclusion, that the methods and data are not robust enough to serve as a standard method for pharmaco-vigilance (12). However, there are some cases, where this may be useful for signal detection or signal conformation. It was however, strongly recommended to not rely only on social media research but instead, to always confirm findings through other means.
- Other examples exist where sentiment analysis has revealed information on people's attitudes and motivations (e.g. with tobacco use of all sorts (14,15), HPV vaccination (16)) with the purpose to inform public health policies or communication. One study analysed how well a new FDA policy relating to tobacco risk warnings in advertisement in social media was implemented or which impact it had on social media campaigns (17).
- Two publications showcase examples of how one specific platform (Inspire) can contribute to better information sourced directly from the patient community. Both studies promote the use of this platform for such research to inform the FDA on issues such as off label drug use (18) or adverse event identification (11)
- Quite a few examples exist where pharmaceutical industry has used SMR to better understand patient experiences and needs and to use this information in the early dialog processes with HTA agencies to support the choice of endpoints (19–22). How much these data are in fact used by different HTA agencies for informing their decisions, is unclear.

Our conclusion is, that many potential applications of social media research in healthcare policy making and implementation or communication are emerging, but not much of this emerging evidence is currently used as state of the art in HTA. No example has been identified, where the HTA agency has directly commissioned a specific piece of social media research.

**References:**

1. JUNG J, BIR C, WIDMAR NO, SAYAL P. Initial Reports of Foodborne Illness Drive More Public Attention Than Do Food Recall Announcements. J Food Prot [Internet]. 2021 Feb 26 [cited 2021 Sep 13];84(7):1150–9. Available from: https://doi.org/10.4315/JFP-20-383

2. Fadaee N, Huynh D, Towfigh S. #Mesh: Social Media and Its Influence on Perceptions in Hernia Repair. Am Surg [Internet]. 2020 Oct [cited 2021 Sep 13];86(10):1351–7. Available from: https://search.ebscohost.com/login.aspx?direct=true&db=mdc&AN=33103471&site=ehost-live

3. Lee C, St Clair C, Merenda CC, Araojo CR, Ray S, Beasley D, et al. Assessment of public and patient online comments in social media and food and drug administration archival data. Res Soc Adm Pharm [Internet]. 2020 Jul 1 [cited 2021 Sep 13];16(7):967–73. Available from: https://www.sciencedirect.com/science/article/pii/S155174111930395X

4. Pierce CE, Bouri K, Pamer C, Proestel S, Rodriguez HW, Van Le H, et al. Evaluation of Facebook and Twitter Monitoring to Detect Safety Signals for Medical Products: An Analysis of Recent FDA Safety Alerts. Drug Saf [Internet]. 2017 Apr 1 [cited 2021 Sep 14];40(4):317–31. Available from: https://doi.org/10.1007/s40264-016-0491-0

5. Seifert HA, Malik RE, Bhattacharya M, Campbell KR, Okun S, Pierce C, et al. Enabling social listening for cardiac safety monitoring: Proceedings from a drug information association–cardiac safety research consortium cosponsored think tank. Am Heart J [Internet]. 2017 Dec 1 [cited 2021 Sep 14];194:107–15. Available from: https://www.sciencedirect.com/science/article/pii/S0002870317302545

6. Rausch C for DE and. CDER Conversation: Monitoring Social Media to Better Understand Drug Use Trends. FDA [Internet]. 2018 [cited 2021 Sep 13]; Available from: https://www.fda.gov/drugs/news-events-human-drugs/cder-conversation-monitoring-social-media-better-understand-drug-use-trends

7. Beasly D, Lee C, Merenda C, Toebe T, Boudreaux J. FDA Science of Engagement: Qualitative Exploration of Unstructured FDA Data Sources and Diabetes Communication on Facebook. In 2018. Available from: http://dx.doi.org/10.1016/j.japh.2018.04.004

8. Chen X, Faviez C, Schuck S, Lillo-Le-Louët A, Texier N, Dahamna B, et al. Mining Patients’ Narratives in Social Media for Pharmacovigilance: Adverse Effects and Misuse of Methylphenidate. Front Pharmacol [Internet]. 2018 [cited 2021 Sep 13];9:541. Available from: https://www.frontiersin.org/article/10.3389/fphar.2018.00541

9. Convertino I, Ferraro S, Blandizzi C, Tuccori M. The usefulness of listening social media for pharmacovigilance purposes: a systematic review. Expert Opin Drug Saf [Internet]. 2018 Nov 2 [cited 2021 Apr 15];17(11):1081–93. Available from: https://www.tandfonline.com/doi/full/10.1080/14740338.2018.1531847

10. The Cardiology Advisor [Internet]. 2019 [cited 2021 Sep 13]. Social Monitoring: How the FDA Mined Social Media to Gain Diabetes Insights. Available from: https://www.thecardiologyadvisor.com/home/topics/metabolic/social-monitoring-how-the-fda-mined-social-media-to-gain-insights-about-patients-with-diabetes/

11. Timimi F, Ray S, Jones E, Aase L, Hoffman K. Patient-Reported Outcomes in Online Communications on Statins, Memory, and Cognition: Qualitative Analysis Using Online Communities. J Med Internet Res [Internet]. 2019 Nov 28 [cited 2021 Sep 13];21(11):e14809. Available from: https://www.ncbi.nlm.nih.gov/pmc/articles/PMC6908973/

12. van Stekelenborg J, Ellenius J, Maskell S, Bergvall T, Caster O, Dasgupta N, et al. Recommendations for the Use of Social Media in Pharmacovigilance: Lessons from IMI WEB-RADR. Drug Saf [Internet]. 2019 Dec 1 [cited 2021 Sep 13];42(12):1393–407. Available from: https://doi.org/10.1007/s40264-019-00858-7

13. Golder S, Smith K, O’Connor K, Gross R, Hennessy S, Gonzalez-Hernandez G. A Comparative View of Reported Adverse Effects of Statins in Social Media, Regulatory Data, Drug Information Databases and Systematic Reviews. Drug Saf [Internet]. 2021 Feb 1 [cited 2021 Sep 13];44(2):167–79. Available from: https://doi.org/10.1007/s40264-020-00998-1

14. Lyu JC, Luli GK. Understanding the Public Discussion About the Centers for Disease Control and Prevention During the COVID-19 Pandemic Using Twitter Data: Text Mining Analysis Study. J Med Internet Res [Internet]. 2021 Feb 9 [cited 2021 Sep 13];23(2):e25108. Available from: https://www.jmir.org/2021/2/e25108

15. Majmundar A, Allem JP, Cruz TB, Unger JB, Pentz MA. Twitter Surveillance at the Intersection of the Triangulum. Nicotine Tob Res [Internet]. 2021 May 6 [cited 2021 Sep 13];(ntab085). Available from: https://doi.org/10.1093/ntr/ntab085

16. Luisi MLR. From bad to worse II: Risk amplification of the HPV vaccine on Facebook. Vaccine [Internet]. 2021 Jan 8 [cited 2021 Sep 16];39(2):303–8. Available from: https://www.sciencedirect.com/science/article/pii/S0264410X20315401

17. Laestadius LI, Wahl MM, Vassey J, Cho YI. Compliance With FDA Nicotine Warning Statement Provisions in E-liquid Promotion Posts on Instagram. Nicotine Tob Res [Internet]. 2020 May 20 [cited 2021 Sep 13];22(10):1823–30. Available from: https://www.ncbi.nlm.nih.gov/pmc/articles/PMC7542640/

18. Nikfarjam A, Ransohoff JD, Callahan A, Polony V, Shah NH. Profiling off-label prescriptions in cancer treatment using social health networks. JAMIA Open [Internet]. 2019 Jul 22 [cited 2021 Sep 13];2(3):301–5. Available from: https://www.ncbi.nlm.nih.gov/pmc/articles/PMC6824514/

19. Booth A, Bell T, Halhol S, Pan S, Welch V, Merinopoulou E, et al. Using Social Media to Uncover Treatment Experiences and Decisions in Patients With Acute Myeloid Leukemia or Myelodysplastic Syndrome Who Are Ineligible for Intensive Chemotherapy: Patient-Centric Qualitative Data Analysis. J Med Internet Res [Internet]. 2019 Nov 22 [cited 2021 Sep 13];21(11):e14285. Available from: https://www.jmir.org/2019/11/e14285

20. Mullins A, Medi S, Cook N, Sloesen B, Prince C, Anand S, et al. Generating Patient Insights In Dry Eye Disease With A Social Media Listening Study. Value Health [Internet]. 2017 Oct 1 [cited 2019 Apr 8];20(9):A807. Available from: https://doi.org/10.1016/j.jval.2017.08.2408

21. Patalano F, Gutzwiller FS, Shah B, Kumari C, Cook NS. Gathering Structured Patient Insight to Drive the PRO Strategy in COPD: Patient-Centric Drug Development from Theory to Practice. Adv Ther [Internet]. 2020;37(1):17–26. Available from: https://link.springer.com/article/10.1007/s12325-019-01134-x

22. Cook NS, Cave J, Holtorf AP. Patient Preference Studies During Early Drug Development: Aligning Stakeholders to Ensure Development Plans Meet Patient Needs. Front Med [Internet]. 2019 [cited 2020 May 19];6(Art. 82):17. Available from: https://www.frontiersin.org/articles/10.3389/fmed.2019.00082/full

1. A docket is a repository through which the public can submit electronic and written comments on specific topics to U.S. federal agencies such as FDA. [↑](#footnote-ref-1)
